# Psychological Well-Being and Mental Health in Caribbean Communities in Light of the Methodological Triangulation of the Classical Approach and Network Analysis

**DOI:** 10.3390/jcm15041416

**Published:** 2026-02-11

**Authors:** Jorge E. Palacio-Sañudo, María Yaquelin Expósito-Concepción, Diana Carolina Consuegra Cabally, María del Carmen Amaris Macías, Ana Liliana Ríos-García

**Affiliations:** 1 Department of Psychology, Universidad del Norte, Barranquilla 080001, Colombia; jpalacio@uninorte.edu.co (J.E.P.-S.); mamaris@uninorte.edu.co (M.d.C.A.M.); 2 Department of Nursing, Universidad del Norte, Barranquilla 080001, Colombia; 3 Department of Public Health, Universidad del Norte, Barranquilla 080001, Colombia; dcabally@uninorte.edu.co (D.C.C.C.); ariosg@uninorte.edu.co (A.L.R.-G.)

**Keywords:** psychological well-being, network analysis, methodological triangulation, mental health, social support

## Abstract

**Background/Objectives:** This study examines psychological well-being and mental health in Caribbean Colombian urban populations through methodological triangulation, integrating traditional statistical analysis with network analysis to develop a comprehensive understanding of protective and risk factors. **Methods:** A cross-sectional study was conducted with 412 participants from Barranquilla and Cartagena. Instruments included Ryff’s Psychological Well-being Scale, Keyes’ Social Well-being Scale, Self-Reporting Questionnaire (SRQ), family APGAR, and perceived social support scales. Data were analyzed using correlational analysis, multiple regression models, and network analysis to achieve methodological triangulation. **Results:** Traditional analysis revealed that social acceptance (β = −0.248), negative emotions (β = −0.268), and family crises (β = 3.272) were significant predictors, explaining 42.2% of mental health variance. Network analysis confirmed these findings through centrality measures, showing social acceptance and social coherence as central nodes. The triangulation between methods validated four integrative hypotheses: differential perceived social support, social coherence as a culturally sensitive protective factor, social support as moderator/mediator of family crises, and the autonomy paradox in collectivist contexts. Notably, the autonomy paradox hypothesis was not empirically supported; autonomy showed a neutral or slightly protective profile, indicating possible cultural adaptation in these urban settings. **Conclusions:** Methodological triangulation between traditional and network approaches provides evidence for multidimensional well-being models in Caribbean Colombian contexts. Social acceptance and family functionality emerge as central protective factors, while family crises constitute primary risk factors. The convergence between analytical methods strengthens the validity of findings and suggests the need for culturally adapted interventions that consider the specificity of collectivist urban contexts in the Colombian Caribbean.

## 1. Introduction

The study of psychological well-being has evolved from unidimensional approaches focused on subjective happiness toward comprehensive perspectives that consider multiple facets of human development [1]. Carol Ryff’s model of psychological well-being, originally developed in Western contexts, has proven particularly relevant by incorporating both individual and relational aspects within its six-dimensional structure. Research such as that conducted by Díaz et al. [2] has shown that these dimensions: self, acceptance, positive relations, autonomy, environmental mastery, personal growth, and purpose in life require cultural adaptations to maintain ecological validity in Latin American contexts, particularly in collectivist societies where the constructs of autonomy and interpersonal relationships acquire nuances different from those originally described in the Anglo-Saxon literature.

This perspective is complemented by Keyes’ model of social well-being [3], which expands the theoretical framework by incorporating five fundamental social dimensions: integration, acceptance, contribution, actualization, and social coherence. As Keyes [4] notes, in cultures such as that of the Colombian Caribbean, where family and community networks play a central role, mental health is sustained by collective or group dynamics. Studies conducted in the Colombian Caribbean have shown that these social dimensions of well-being display stronger correlations with mental health indicators than purely individual aspects [5]. This suggests that, in cultural contexts such as ours, theoretical models should prioritize the relational and community components of psychological well-being.

However, the complexity of these phenomena in our setting requires moving beyond traditional methodological approaches. While correlational analyses and multiple regression models [6] have provided valuable evidence on linear relationships between variables, they are insufficient to capture the dynamic and relational nature of well-being in specific cultural contexts. In such cases, network analysis [7] emerges as an alternative that can identify patterns of interconnection among variables beyond traditional methods. This approach is particularly relevant for studying how dimensions such as autonomy or positive relations are articulated in unique ways within collectivist cultures.

In this regard, methodological triangulation between these approaches becomes an important strategy for advancing the understanding of psychological well-being in such contexts. As Joshanloo et al. [8] point out, the convergence of diverse methods not only increases the validity of findings but also makes it possible to capture the complexity of evolving psychosocial phenomena. In the Colombian Caribbean, where deeply rooted cultural traditions coexist with accelerated processes of urban modernization, this integrative approach is particularly pertinent. Previous studies in the region [9] have identified unique patterns in the configuration of well-being that do not align with expectations derived from traditional theoretical models, reinforcing the need to adopt innovative methodological perspectives.

Moreover, recent research in cities such as Barranquilla and Cartagena [10] has documented how the post-pandemic context, along with processes of internal migration and social mobility, is reshaping the determinants of psychological well-being and mental health. These changes generate tensions between traditional values of family interdependence and the new demands for individual autonomy inherent to urban environments. In this regard, Naberushkina et al. [11] note that, from the perspective of young people, although they recognize the family as crucial in people’s lives, the traditional view of the family has been changing, with an emerging individualistic approach to family life that undermines its capacity to meet emotional needs for love, care, and personal support. Hence, they emphasize the need for policies that support the restoration of the family as a guarantor of the well-being of its members.

As Woodman & Ross [12] point out, although the importance of family ties and relationships for adolescents’ mental health is widely recognized, such relationships can have either a positive or negative impact on young people, closely linked to the degree of family functionality.

Several studies have shown a positive correlation between family functionality and psychological well-being in adults. This means that better family functionality is directly related to higher levels of psychological well-being [13]. Similarly, a study conducted among high school students in Ecuador found that higher family functionality was associated with greater subjective well-being [14].

In light of this complexity, the present study seeks to overcome the limitations of previous research through an innovative methodological approach that combines traditional analyses with the potential of network analysis.

To this end, protective and risk factors for mental health were identified through correlational analyses and multiple regression models. The network structure of psychological well-being and mental health variables was examined using network analysis, triangulation of findings between traditional methods and network analysis was carried out to validate the convergence of results, and an integrative model was developed incorporating the cultural specificity of the Colombian Caribbean context.

The following Integrative Hypotheses are presented:

**H1.**  *Differential Perceived Social Support by Source and Cultural Context*.

Among urban young adults in the Colombian Caribbean, support from friends (horizontal) is expected to have a greater impact on well-being and mental health than family support (vertical), due to processes of autonomy and changes in support networks. This hypothesis is grounded in the buffering role of social support against stress [15] and in the relevance of support sources for social well-being [3]. Evidence from Latin America confirms the importance of peer support among urban youth [5,9], and systematic reviews highlight its differential effect on mental health [16].

**H2.**  *Social Coherence as a Culturally Sensitive Protective Factor*.

In urban contexts of the Colombian Caribbean, characterized by high complexity, cultural diversity, and uncertainty, social coherence understood as the ability to comprehend and make sense of social dynamics acts as a protective factor against psychological distress. This concept, a cornerstone of social well-being according to [3], has shown relevance in scenarios marked by high mobility and inequality [8]. A systematic review in multicultural contexts confirms its role in resilience and mental health [17], and regional studies corroborate this finding in the Colombian Caribbean [5].

**H3.**  *Social Support as a Moderator/Mediator of Family Crises and Mental Health*.

Perceived social support acts as a mediator or moderator between family crises and mental health, buffering the negative effect of stressful events on psychological well-being, particularly in contexts of vulnerability and social change. This protective effect is explained by the stress-buffering model [15] and the theories of Keyes [4] and Ryff [1]. Latin American evidence confirms this role in populations affected by family and social crises [18,19], and a systematic review supports its function in the face of adverse life events [20,21].

**H4.**  *The High-Autonomy Paradox in Collectivist Contexts*.

In urban contexts of the Colombian Caribbean, high levels of autonomy may be associated with greater psychological distress due to the tension between individualistic values and the collectivist expectations characteristic of the region. Although autonomy is a key element in Western theories of well-being [1], research in Latin America shows that it can generate role conflicts and isolation in collectivist cultures [5,20]. A systematic review confirms that its protective effect depends on the cultural context [8].

## 2. Materials and Methods

To test the hypotheses, a cross-sectional descriptive–correlational design was implemented with the aim of examining the relationships among variables of psychological well-being, social support, family functionality, and mental health in a sample from urban populations in the Colombian Caribbean. The study integrated traditional statistical analyses with network analysis to achieve methodological triangulation and to compare the validity of the findings using different analytical strategies.

### 2.1. Participants

The study was conducted in the Caribbean region of Colombia, specifically in the Department of Atlántico (Barranquilla District) and the Department of Bolívar (Cartagena District), in low socioeconomic status neighborhoods, using accessibility and snowball sampling.

Two clusters were selected one in each district/municipality and the instruments were administered in the selected area by previously trained personnel. Households without family members over the age of 18 were excluded. Recruitment for participation was carried out through educational institutions and foundations with representativeness in the study context, which is essential in community-based research, as it facilitates access and promotes the active participation of families.

Convenience and snowball sampling constitute an appropriate strategy in community-based research and studies involving vulnerable populations, due to the access difficulties, institutional mistrust, and geographical dispersion that often characterize these groups [22].

A non-probabilistic convenience and snowball sampling method was used, considered appropriate for community-based studies involving vulnerable families, as it facilitates access to hard-to-reach participants and promotes the inclusion of subgroups often excluded from probabilistic sampling, thereby preserving the contextual validity of the information. Moreover, this approach is regarded as relevant and ethically responsible when the aim is to understand social, family, or community phenomena from an exploratory and interpretive perspective, rather than to estimate population parameters.

No a priori power calculation was performed due to the exploratory nature of the study and the sampling strategy. The findings were interpreted by prioritizing effect sizes and confidence intervals.

The study was conducted following the recommendations of the STROBE guidelines (Strengthening the Reporting of Observational Studies in Epidemiology) to ensure transparency and quality in the reporting of observational research. Compliance with the 22 items corresponding to the sections on title, abstract, introduction, methodology, results, and discussion was verified. The review showed 90% adherence to the applicable criteria. The complete STROBE checklist, with cross-references to each item in the manuscript, is included as Supplementary Materials.

The sociodemographic characteristics of the sample showed a gender distribution of 79 men (19.2%) and 333 women (80.8%). The mean age was 40.27 years (SD = 14.78, range 18–65 years). Regarding educational level, 13 participants (3.2%) had no formal education; the most represented levels were completed secondary education (136 participants, 33%), incomplete secondary education (84 participants, 20.4%), and technical education (90 participants, 21.8%). (Table 1).

### 2.2. Instruments

The instruments were administered at times agreed upon with the participants to avoid fatigue effects and the possibility of falsified responses. Data collection was conducted in two separate sessions with a minimum interval of two days, following the signing of informed consent.

Ryff’s Psychological Well-Being Scale: The Spanish adaptation validated by Díaz et al. [2] was used, which assesses six dimensions of psychological well-being across 29 items: self-acceptance, positive relations, autonomy, environmental mastery, personal growth, and purpose in life. The scale uses a 6-point Likert format (1 = strongly disagree to 6 = strongly agree). The instrument demonstrated good internal consistency in the evaluated sample (α = 0.80).

Keyes’ Social Well-Being Scale [3]: The adapted version was used, which assesses five dimensions of social well-being through 25 items: social integration, social acceptance, social contribution, social actualization, and social coherence. It uses a 5-point Likert scale (1 = never to 5 = always). This instrument showed good internal consistency in the evaluated sample (α = 0.82).

The subjective well-being scale by Diener (5 items), specifically assessing the life satisfaction dimension, was also used. It employs a Likert-type response scale ranging from 1 (Strongly disagree) to 5 (Strongly agree) and showed acceptable internal consistency in the evaluated sample (α = 0.78) [23].

Positive and Negative Affect Schedule (PANAS) [24]: This instrument measured positive and negative emotions using a 12-item validated version for Latin American populations, with responses given on a 5-point Likert scale (1 = never to 5 = always). Internal consistency for the Positive Affect dimension was good (α = 0.83), while for the Negative Affect dimension it was acceptable (α = 0.725).

Self-Reporting Questionnaire (SRQ): The SRQ-30 validated for the Colombian population was used, consisting of 30 items [21,25], allowing the screening of common mental health problems, including anxiety symptoms, depressive symptoms, and alcohol-related problems. Although the SRQ includes an item aimed at exploring signs of psychotic symptoms, in this study we considered it inappropriate to report this indicator independently. The main reason is that the SRQ, being a screening instrument, identifies the possible presence of psychosis based on a single affirmative response, which may lead to overestimation or misinterpretation of such manifestations in non-clinical populations.

Following methodological recommendations for the use and interpretation of the SRQ, we chose to focus on global indicators and domains with stronger empirical support, such as anxiety, depression, and somatization, avoiding diagnostic conclusions derived from a single item. The instrument showed acceptable internal consistency in the selected sample (KR-21 = 0.71). This result reinforced the decision not to include that component in the analyses or in the presentation of results. We believe this choice contributes to maintaining methodological coherence and interpretative validity in the study, while acknowledging that the SRQ is a screening tool, not a diagnostic instrument.

Multidimensional Scale of Perceived Social Support: Three subscales were administered, totaling 12 items, which assess perceived social support from family, friends, and significant others. Each subscale uses a 7-point Likert format, and reliability coefficients for the sample were above α = 0.85 [26].

Family APGAR: This tool, developed by Smilkstein [27] in 1978, consists of five closed-ended questions in a self-administered questionnaire that assesses the family functioning as perceived by the respondent, allowing the suspicion of dysfunction but not its diagnosis. Each question can be scored from 0 to 4 (never, almost never, sometimes, almost always, and always); the questionnaire yields scores from 0 to 20, where scores of 9 or less indicate severe family dysfunction, 10 to 13 indicate moderate family dysfunction, 14 to 17 indicate mild family dysfunction, and scores above 18 indicate good family functioning.

Normative and Non-Normative Family Crises: A specific instrument or ad-hoc questionnaire that assesses situations such as violent death, natural death, illness, separation, leaving the household, arrival of a new member, starting school, expulsion from school, unemployment, relationship problems, retirement, economic changes, pregnancy, adoption, and infidelity.

### 2.3. Statistical Analyses

The study integrated traditional statistical analyses with network analysis to achieve methodological triangulation and to compare the validity of the findings using different analytical strategies [28].

The database was prepared in MS Excel, where the extracted data were coded, and an initial filtering process was performed. In IBM SPSS Statistics v.29, the variables were analyzed using absolute and relative frequencies in percentages or with measures of central tendency and dispersion, after verifying the normality of the distribution.

Preprocessing included a thorough review of data quality through cleaning procedures to remove erroneous or inconsistent data. Outliers were identified and analyzed to ensure the integrity of the analysis. In addition, the statistical assumptions required for the applied models were verified, such as linearity in the logit for logistic regression, using specific tests, to ensure the validity of the results.

To handle missing data, the multiple imputation technique was applied to the variable Family APGAR, which showed the highest percentage of missing values, in order to avoid bias in the analyses and improve statistical validity. In the case of the variable with a lower percentage of missing data (Individual life stage), those observations were excluded due to their low incidence.

### 2.4. Network Analysis

The items in our scale are rated on a 5-point Likert scale. To estimate the network, we first calculated the polychoric correlation matrix using the cor_auto function integrated in JASP and the bootnet package. The literature indicates that when data are ordinal, the Gaussian Graphical Model (GGM) should be based on polychoric correlations in order to preserve the ordinal nature of the data [29]. This procedure avoids assuming multivariate normality. Recent studies have shown that polychoric correlations allow for a more accurate representation of ordinal variables [29]. The manuscript now specifies the use of this type of correlation and includes a note in the methods section indicating that Spearman correlation was discarded after verifying that the polychoric matrix was positive definite.

The network selection was performed using the graphical LASSO (Least Absolute Shrinkage and Selection Operator) algorithm, minimizing the EBIC (Extended Bayesian Information Criterion). The EBIC incorporates the γ parameter to control the penalization of model complexity; it is recommended to set it between 0 and 0.5 [29]. In our analysis, γ = 0.5 was used, a value that favors more parsimonious models and reduces the likelihood of false positives.

The model was selected by identifying the minimum EBIC value among networks generated with different λ values [29].

According to Epskamp et al. [30], applying significance tests to an already regularized network is unnecessary, as the presence or absence of an edge results from the regularization process [30]. Therefore, in the revised version, we removed the Bonferroni threshold for edge weights and focused on interpreting the existing connections and their confidence intervals [29].

A nonparametric bootstrap with 1000 replications was performed to obtain 95% confidence intervals for the edge weights [31]. Edges with narrower intervals are interpreted as more stable.

A case-dropping bootstrap was applied to calculate the centrality stability coefficient (CS), which estimates the maximum proportion of participants that can be excluded without altering the order of node centrality [31]. A CS > 0.25 is considered acceptable, and values above 0.50 are regarded as optimal. In our data, the strength index obtained a CS of 0.36, while betweenness and closeness showed values below 0.25; therefore, only the strength centrality was interpreted.

The factor loadings from an exploratory factor analysis were correlated with the strength indices of the network. A moderate positive correlation was found, suggesting convergence between both approaches.

We calculated Kendall’s (τ) and Spearman’s coefficients between the ranking of strength and the ranking of factor loadings. These coefficients are presented in a table, allowing for a quantitative assessment of the correspondence between methods [31].

The results of traditional analyses were systematically compared with the findings from network analysis to identify convergences and divergences. The consistency of protective and risk factors identified by both methods was evaluated.

### 2.5. Ethical Considerations

This study complied with the provisions of Resolution 8430 of 1993, which regulates health research in Colombia, and with the Helsinki Declaration. It was approved by the Research Ethics Committee in the Health Area of Universidad del Norte under Minutes No. 264 of 2022.

## 3. Results

### 3.1. Psychological and Social Well-Being

Analysis of the dimensions of psychological well-being according to Ryff’s scale showed that autonomy had the highest mean score (M = 24.79, SD = 5.48), followed by purpose in life (M = 24.53, SD = 5.49) and environmental mastery (M = 23.12, SD = 5.12). Conversely, positive relations had the lowest mean score (M = 15.67, SD = 4.87), suggesting challenges in building satisfactory interpersonal relationships in urban contexts of the Colombian Caribbean (Table 2).

Table 2 also presents Keyes’ social well-being results, where social contribution obtained the highest mean score (M = 20.01, SD = 5.93), indicating that participants perceive themselves as contributing positively to their community. However, social coherence showed the lowest mean score (M = 11.94, SD = 4.06), which may reflect difficulties in understanding social dynamics in contexts of urban complexity.

Subjective well-being showed a mean life satisfaction score of 17.12 (SD = 5.49), while perceived social support results indicated that family support had the highest mean score (M = 22.53, SD = 7.22), followed by friend support (M = 21.11, SD = 7.37) and support from significant others (M = 17.93, SD = 8.89).

### 3.2. Prevalence of Mental Health Problems

Table 3 shows an overall prevalence of mental health problems of 11.33% in the total sample, while alcohol-related problems reached 14.46%. Cartagena shows higher prevalences of mental health problems, psychosis, and alcoholism compared to Barranquilla.

Table 4 shows that 36.5% of families present good functioning, 33.1% mild dysfunction, 8.4% moderate dysfunction, and 9.0% severe dysfunction. In caregiver burden, the absence of overload predominates, although there is a fraction with intense burden that requires attention.

In family functionality, a mixed picture is observed: although good function and mild dysfunction predominate, around 1 in 4 families present moderate or severe dysfunction, which may affect the mental health and well-being of their members.

### 3.3. Correlational Analysis: Protective and Risk Factors

Spearman correlations revealed significant patterns between the dimensions of well-being and mental health indicators. Table 4 shows that social acceptance emerged as the strongest protective factor, displaying significant negative correlations with mental health screening (r = −0.405, *p* < 0.01) and mental health problems (r = −0.362, *p* < 0.01). As Keyes states, “social acceptance reflects trust, acceptance, and positive attitudes toward others—indicators of mental health”.

Social coherence also exhibited significant protective associations (r = −0.292 with mental health screening, r = −0.279 with mental health problems), confirming its role as a mediator between understanding the social environment and psychological well-being. Subjective well-being showed protective correlations as well (r = −0.215 with screening, r = −0.256 with mental health problems), validating the importance of affective balance for mental health (Table 5).

Regarding perceived social support, Table 4 shows that friendship support displayed stronger protective correlations (r = −0.212 with mental health screening, r = −0.208 with mental health problems) compared to family support (r = −0.167 with mental health problems). This pattern suggests differential effects depending on the source of support, confirming the hypothesis of culturally contextualized differential social support.

Family functioning, as measured by the APGAR, showed significant negative correlations with mental health problems (r = −0.188, *p* < 0.05), whereas the Zarit Burden Interview exhibited stronger correlations (r = −0.290 with screening, r = −0.268 with mental health problems) (Table 5).

### 3.4. Bivariate Analysis of Sociodemographic Variables and Mental Health

The bivariate analysis of demographic variables and mental health (SQR) is presented in Table 6. The bivariate analysis identified specific sociodemographic risk factors. Family structure showed significant associations (*p* < 0.001), with single-parent families presenting a higher risk (7 out of 15 cases at high risk). Couple problems emerged as a significant risk factor (*p* < 0.001), with 7 out of 19 cases at risk.

Social support variables showed consistent protective effects. The absence of support from significant others was associated with higher risk (9 out of 20 cases), while its presence was protective (7 out of 158 cases at high risk). Similar patterns were observed for family support and support from friends.

### 3.5. Traditional Predictive Models

The linear regression model showed a good fit (adjusted R^2^ = 0.299; F (10,359) = 16.741; *p* < 0.001; Durbin–Watson = 1.86). The significant predictors before correction were: gender, family functioning (APGAR), social coherence, city, relationship problems, negative emotions, friend support, and family support.

After applying the False Discovery Rate (FDR) correction (Benjamini–Hochberg, Q = 0.05), the variables gender, family functioning, social coherence, city, relationship problems, negative emotions, and friend support remained significant. In contrast, the predictors social acceptance, family structure, and family support did not retain statistical significance under this criterion (Table 7).

### 3.6. Binary Logistic Regression Model

The binary logistic regression model was globally significant (χ^2^ (15) = 82.16, *p* < 0.001), showing a moderate explanatory capacity (Nagelkerke R^2^ = 0.393). The model fit was adequate (Hosmer–Lemeshow χ^2^ = 2.43, *p* = 0.965), and the predictive accuracy reached 91.4% correct classification (98.2% for absence and 38.1% for presence of mental health problems).

Before correction, the statistically significant predictors were social coherence, negative emotions, friend support, city, and relationship problems. After applying the FDR correction, the predictors that remained significant were social coherence, negative emotions, city, and relationship problems, while friend support lost statistical significance (Table 8).

### 3.7. Network Analysis: General Structure of the Network

An Appendix includes the edge confidence interval plots and centrality difference plots, as well as the CS coefficient table, following the recommendations of Forbes et al. [31].

**Figure 1:**
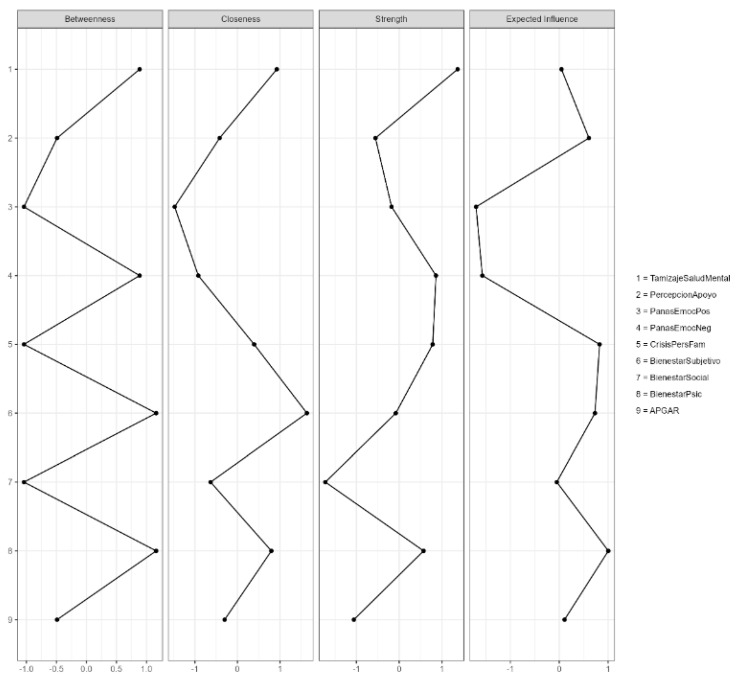
Centrality measures. Caribbean Region of Colombia (Departments of Atlántico and Bolívar), 2023–2024. 1—Psychological Mental Health Screening (Tamizaje Salud Mental), 2—Perceived Support (Percepción Apoyo), 3—PANAS Positive Affect (Emociones Positivas), 4—PANAS Negative Affect (Emociones Negativas), 5—Personal/Familial Crisis (Crisis Personales Familiares), 6—Well-being (Bienestar Subjetivo), 7—Social Well-being (Bienestar Social), 8—Subjective Well-being (Bienestar Psicológico), 9—Family Functionality (APGAR).

Centrality analysis showed that Psychological Well-being and Subjective Well-being occupied central positions in the network, with higher betweenness and closeness values, indicating their integrative role among the other variables. Mental Health Screening presented the highest strength, reflecting strong connections with various dimensions of well-being and personal/familial crisis. In contrast, Social Well-being and Positive Affect recorded the lowest values, indicating lower centrality. Overall, the network exhibited a moderately integrated structure, in which psychological and subjective well-being act as key nodes in the interaction between family functionality, perceived support, and mental health (Figure 1; Appendix Table A9).

The plots display the standardized values for betweenness, closeness, strength, and expected influence. Psychological and Subjective Well-being show the highest centrality values, indicating their integrative role within the network, while Social Well-being and Positive Affect exhibit the lowest scores.

**Figure 2:**
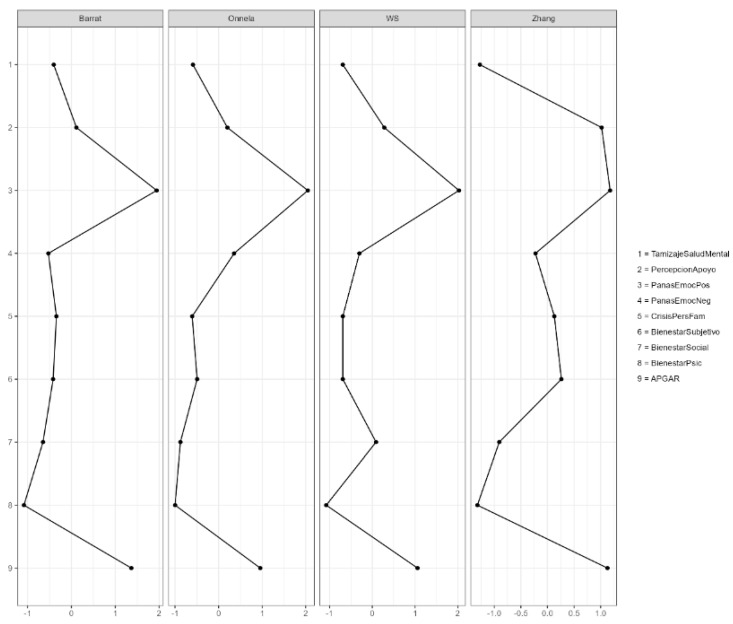
Clustering measures. Caribbean Region of Colombia (Departments of Atlántico and Bolívar), 2023–2024. 1—Psychological Mental Health Screening (Tamizaje Salud Mental), 2—Perceived Support (Percepción Apoyo), 3—PANAS Positive Affect (Emociones Positivas), 4—PANAS Negative Affect (Emociones Negativas), 5—Personal/Familial Crisis (Crisis Personales Familiares), 6—Well-being (Bienestar Subjetivo), 7—Social Well-being (Bienestar Social), 8—Subjective Well-being (Bienestar Psicológico), 9—Family Functionality (APGAR).

The clustering coefficient analysis (Figure 2) showed notable variability among the variables according to the Barrat, Onnela, WS, and Zhang indices. Positive Affect (PANAS) and Family Functionality (APGAR) exhibited the highest clustering values across the four measures, indicating strong local interconnections within the network. In contrast, Psychological Well-being, Mental Health Screening, and Personal/Familial Crisis presented the lowest coefficients, suggesting weaker local connectivity and less cohesion around these nodes.

The network graph revealed a moderately connected structure among the nine variables. The most prominent positive association was observed between Personal/Familial Crisis and Mental Health Screening, represented by a thick blue edge, indicating that higher levels of crisis were linked to greater mental health risk. Another relevant positive connection emerged between Family Functionality (APGAR) and Subjective/Psychological Well-being, suggesting that better family relationships are associated with higher levels of psychological adjustment and life satisfaction.

In contrast, a strong negative association was identified between Positive Affect and Negative Affect (red edge), reflecting the emotional polarity within the network. Perceived Support maintained moderate positive links with APGAR and Psychological Well-being, highlighting its role as a social buffer related to mental health protection.

**Figure 3:**
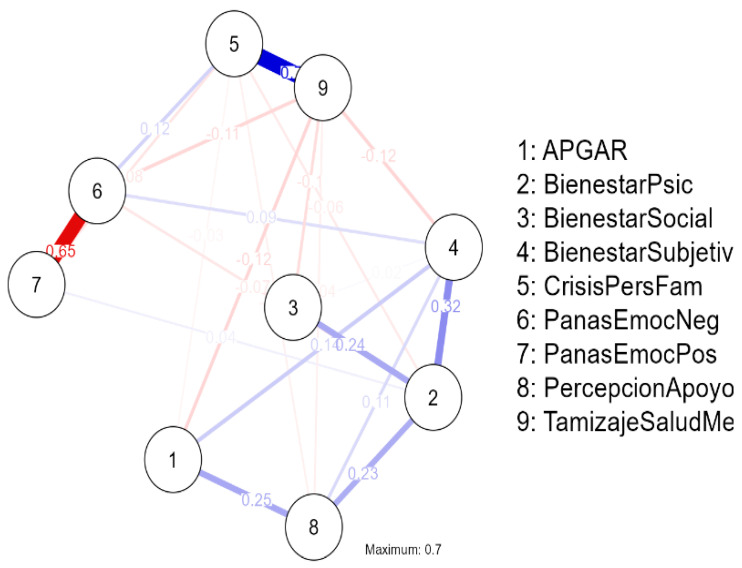
General network graph. Caribbean Region of Colombia (Departments of Atlántico and Bolívar), 2023–2024. 1—Family Functionality (APGAR), 2—Psychological Well-being (Bienestar Psicológico), 3—Social Well-being (Bienestar Social), 4—Subjective Well-being (Bienestar Subjetivo), 5—Personal/Familial Crisis (Crisis Personales Familiares), 6—PANAS Negative Affect (Afecto Negativo), 7—PANAS Positive Affect (Afecto Positivo), 8—Perceived Support (Percepción Apoyo), 9—Mental Health Screening (Tamizaje Salud Mental). Positive correlation is blue and Negative correlation is red.

Overall, the network exhibits two main clusters: one grouping well-being, family functionality, and support, and another linking crisis, negative affect, and mental health screening, indicating differentiated yet interconnected domains of emotional and relational functioning (Figure 3).

### 3.8. Network Analysis of Psychosocial Variables

The estimated network revealed significant relationships among family, emotional, and well-being variables. A strong association was identified between personal/familial crises and mental health screening (r = 0.698), indicating that higher levels of crisis are related to an increased risk of psychological distress. Likewise, a negative relationship was observed between positive and negative emotions (r = −0.650), and positive links between family functionality (APGAR), perceived social support, and subjective well-being, suggesting that family functionality enhances perceived well-being.

**Figure 4:**
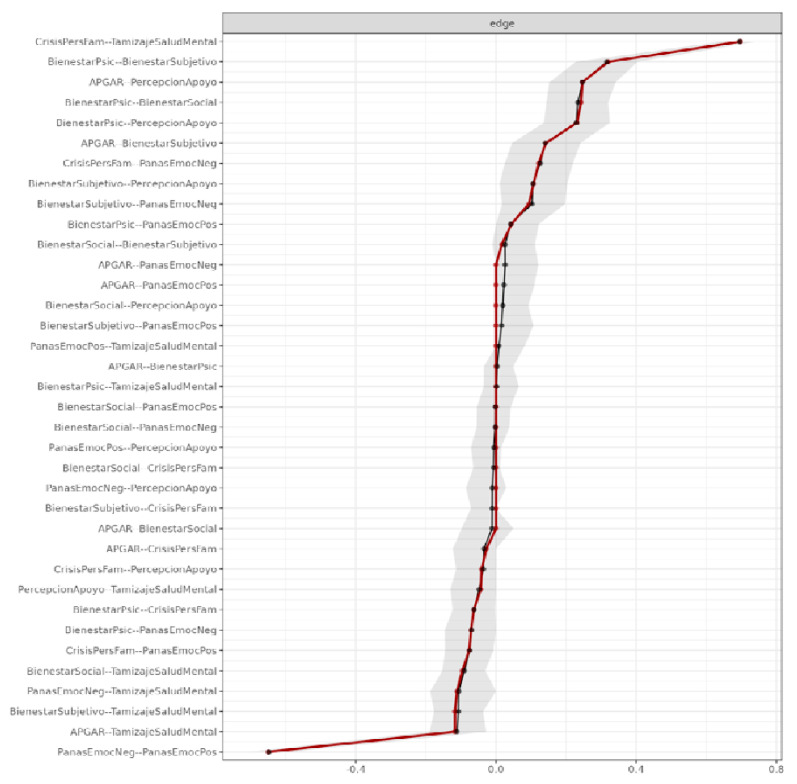
Bootstrapped mean and confidence intervals of edge weights, indicating adequate precision and stability across the network. Black line Bootstrap mean, Red line sample mean.

In the network structure, mental health screening, negative emotions, and family crises showed the greatest connection strength, while psychological and subjective well-being acted as bridge nodes integrating the emotional, social, and family domains. The stability analysis revealed narrow confidence intervals, demonstrating consistency in the most relevant associations (Figure 4).

Overall, the network revealed three main clusters: a well-being cluster (psychological, subjective, and social), an emotional cluster (positive and negative affect), and a risk cluster (family crisis and mental health). Although peripheral, the family functionality (APGAR) variable plays a protective role by strengthening perceived support and overall well-being.

## 4. Discussion

The results provide convergent evidence regarding the determinants of psychological well-being and mental health among urban populations in the Colombian Caribbean. The triangulation between traditional statistical analyses and network analysis revealed consistent patterns that strengthen the validity of the findings and offer a deeper understanding of the complex dynamics of well-being within culturally specific contexts.

The estimated network displayed a structure consistent with the correlational and regression results, identifying three main clusters: one related to well-being (psychological, subjective, and social), another to emotion (positive and negative affect), and a risk cluster (family crises and mental health screening). The strongest associations emerged between personal and family crises and mental health screening, as well as between positive and negative emotions. These relationships reflect the coexistence of interdependent protective and risk factors shaping individuals’ psychological experiences.

Centrality analysis indicated that mental health screening, negative emotions, and family crises were the most influential nodes within the network, functioning as sensitive indicators of psychological distress. Meanwhile, psychological and subjective well-being acted as bridge nodes, integrating emotional, social, and family domains. Although peripheral, family functioning (APGAR) maintained positive connections with perceived support and overall well-being, suggesting an indirect protective role within the system’s global configuration.

### 4.1. Validation of Integrative Hypotheses

**Hypothesis** **1.** *Differential perceived social support by source and cultural context.*

This hypothesis was partially confirmed. Correlational analyses revealed that support from friends had a stronger protective effect on mental health than family support. In the network, this pattern was most evident among subgroups with higher autonomy, particularly urban youth, where peer support exhibited greater centrality. This finding reflects ongoing social transitions in Caribbean urban settings, where young people are shifting from traditional family-based networks to more horizontal social structures. Indeed, horizontal relationships, characterized by equality and reciprocity among peers, are critical to the development of social and emotional competencies, particularly in contexts outside the family setting [32].

**Hypothesis** **2.** *Social coherence as a culturally sensitive protective factor.*

This hypothesis was fully supported. Social coherence showed significant negative correlations with mental health problems and remained a significant predictor in regression models. Within the network, it exhibited high centrality and functioned as an integrative node linking the emotional and social well-being clusters. In complex urban Caribbean settings where traditional and modern dynamics coexist the capacity to make sense of social experience becomes fundamental for psychological stability, consistent with prior studies emphasizing the role of social coherence in resilience and cultural adaptation [8].

**Hypothesis** **3.** *Social support as a moderator/mediator between family crises and mental health.*

The findings support this hypothesis. Bivariate analyses demonstrated that social support significantly reduces the risk of mental health problems, even in the presence of severe family crises. Network analysis further revealed that, among participants experiencing intense family crises, external social support gained greater centrality, suggesting compensatory mechanisms. Family crises related to separation were identified as the main risk factor; however, their impact was significantly mitigated by robust social support, consistent with the stress-buffering model proposed by Cohen and Wills [15].

**Hypothesis** **4.** *The paradox of high autonomy in collectivist contexts.*

This hypothesis was not confirmed. Contrary to expectations, autonomy was not negatively associated with mental health; instead, it showed a neutral or slightly protective profile. This suggests that urban modernization processes in the Colombian Caribbean have enabled a cultural adaptation allowing the coexistence of individual autonomy and traditional collectivist values without generating psychological conflict. This pattern contrasts with findings from more traditional societies, where high autonomy often leads to cultural dissonance [20].

### 4.2. Convergence Between Classical and Network Analyses

The convergence between the two methodological approaches reinforces both internal and external validity. The main predictors identified, social acceptance, social coherence, and family crises, corresponded to the most central nodes in the network, indicating that both methods capture complementary aspects of the phenomenon: traditional analyses quantify the strength of associations, while network analysis reveals their structural role within the psychosocial system [28].

The clustering patterns observed in the network were consistent with the theoretical dimensions of social, family, and individual well-being described by Keyes [28], confirming the relevance of the multidimensional well-being model in this cultural context. Moreover, variations across city, gender, and life stage were consistent in both approaches, reinforcing that well-being and vulnerability trajectories are shaped by sociodemographic and contextual factors [31,32,33].

### 4.3. Theoretical Implications

The findings make significant contributions to the theoretical development of integrative models of psychological well-being:

Model of Social Well-Being Centrality: Social dimensions—particularly social acceptance and coherence—act as central nodes mediating the relationships among individual, family, and mental health factors [28,31,34].

Model of Social Support Compensation: In the context of family crises, the availability of external social support can reorganize the structure of well-being to maintain psychological stability, consistent with theories of adaptive plasticity [35,36].

Model of Cultural Specificity: The differentiated patterns observed in the Colombian Caribbean—highlighting the centrality of social support and the absence of paradoxical effects of autonomy—underscore the need to adapt well-being models to cultural contexts. Such adaptation enhances both the predictive validity and practical utility of these models for designing localized and culturally sensitive interventions [32,37,38].

### 4.4. Clinical and Intervention Implications

Social Acceptance–Centered Interventions: Group programs promoting inclusion, empathy, and social cohesion could strengthen this key protective factor [39,40,41].

Strengthening Social Coherence: Educational and community-based strategies that foster understanding of social dynamics and the development of coherent collective narratives may enhance psychological resilience [42,43,44].

Differentiated Social Support Interventions: Among urban youth, promoting horizontal peer networks may be more effective than interventions focused exclusively on family relationships [45,46,47].

Preventive Family Interventions: The identification of family crises as the main risk factor underscores the need for prevention programs and early family interventions. Strategies aimed at strengthening family functioning and crisis management can have significant protective effects [48,49,50].

### 4.5. Cultural Considerations for the Colombian Caribbean

The results highlight several sociocultural features that must be considered for interpretation and intervention design:

Cultural adaptation of autonomy, reflecting a functional synthesis between individualistic and collectivist values [51,52].

Centrality of social support as a key resource for coping and well-being promotion.

Flexibility of family structures, reflecting adaptive strengths amid rapid social change.

### 4.6. Study Limitations

As the main limitation, the descriptive nature of the study and the use of a non-probabilistic sampling method within a cross-sectional design involving families in conditions of vulnerability are acknowledged, which restricts statistical representativeness and, consequently, the generalization of the findings. Likewise, the presence of social desirability bias cannot be ruled out, since some responses may have been influenced by the intention to present a more favorable situation than the actual one. Furthermore, the marked gender imbalance in the sample constitutes a significant limitation that should be considered with caution when interpreting the results and their potential generalization.

To mitigate potential biases in the interpretation of the results, several methodological strategies were implemented. First, clear inclusion and exclusion criteria were established to ensure coherence in the selection of the study population. Second, data collection was carried out using standardized instruments administered by previously trained researchers, reducing variability in survey administration and interviewer influence. In addition, stratified analyses were conducted according to sociodemographic variables and municipality, allowing the identification of differential patterns and preventing homogeneous conclusions that could obscure inequities. Triangulation with secondary sources strengthened the consistency of the findings, while transparent reporting of limitations contributed to a critical and contextualized interpretation. Finally, collective and multidisciplinary review of the results reduced the risk of biased interpretations, promoting a more comprehensive and balanced approach.

The cross-sectional and non-probabilistic design limits causal inferences, allowing only the identification of associative patterns rather than causal relationships. Although the predictive and network models yielded consistent results, the directionality of the identified relationships requires longitudinal confirmation.

Future research should incorporate longitudinal designs and probabilistic sampling across diverse urban and rural regions to validate the findings and strengthen their generalizability. Likewise, expanding the sample to include other contexts in the Caribbean and Latin America would enhance the external validity and cultural applicability of the proposed integrative model, aimed at achieving a deeper understanding of the protective and risk factors that influence psychological well-being and mental health in families.

## 5. Conclusions

The study provides evidence on the feasibility and added value of methodological triangulation between traditional statistical analyses and network analysis for the study of psychological well-being and mental health in urban populations of the Colombian Caribbean. The findings offer convergent evidence that strengthens a more detailed understanding of the complex dynamics of well-being in culturally specific contexts.

Social acceptance emerges as the most consistent protective factor, standing out simultaneously in regression models and network analyses, reaffirming its key role in psychosocial integration within collectivist contexts. Social coherence emerges as a culturally sensitive protective factor and as a mediator between individual and social functioning,

Family crises, especially marital separation, are the main risk factor; however, their effect is notably reduced by robust social support, evidencing buffering and adaptive mechanisms. Social support shows differential effects depending on the source, with horizontal support standing out among urban youth, reflecting a cultural adaptation that combines collectivist values with modern demands for autonomy.

In contrast to the autonomy paradox hypothesis, no significant negative relationship with mental health was observed; on the contrary, it showed a neutral or slightly protective effect. This suggests that, in urban and hybrid contexts of the Colombian Caribbean, autonomy can be integrated without generating psychological conflict, in line with recent studies on the cultural adaptation of individual values in collectivist societies.

Methodological triangulation demonstrated strong convergence between traditional analyses and network analysis, strengthening the validity of the results and revealing complementary perspectives on the same phenomena. Network analysis highlighted latent structures and critical nodes not detected by classical methods, as well as differentiated patterns across subgroups, underscoring the heterogeneity of well-being and the need for approaches adapted to sociodemographic and contextual characteristics.

The findings support integrative models of well-being that combine individual and social dimensions, highlighting the centrality of social functioning and proposing mechanisms of social mediation. The validation of hypotheses adapted to the Colombian Caribbean favors the development of culturally sensitive models for Latin American psychology. In addition, the identification of compensatory mechanisms through network analysis provides evidence of the adaptive plasticity of well-being and its role in resilience in the face of adversity.

The results provide guidance for public mental health policies in the Colombian Caribbean and similar sociocultural contexts, prioritizing protective factors such as social acceptance, social coherence, and differential social support. It is recommended to strengthen community networks to moderate the impact of family crises and promote social cohesion, as well as to develop specific family support and education policies to prevent crises and reinforce family functioning.

The findings highlight the need for differentiated policies according to demographic characteristics, prioritizing urban youth, families in crisis, and vulnerable populations. The applied methodological triangulation sets a precedent for research in health psychology that integrates traditional methods and network analysis, demonstrating the potential of hybrid approaches that leverage the strengths of both methodologies.

The comprehensive model developed for the Colombian Caribbean can serve as a reference for comparative studies in other Latin American contexts, strengthening a culturally grounded health psychology. Its clinical application requires interventions that translate evidence into programs for the promotion of well-being and the prevention of mental health problems, adapted to cultural specificities and local strengths.

In summary, this study demonstrates that the integration of traditional approaches and network analysis provides a richer and more nuanced understanding of psychological well-being and mental health, with theoretical, methodological, and practical implications for the field of mental health and well-being in Latin American contexts.

## Figures and Tables

**Table 1:** Sociodemographic characteristics of participants—Caribbean Region of Colombia (Departments of Atlántico and Bolívar), 2023–2024.

Variables	Frequency	Percentage
**City**		
Barranquilla	178	43.2
Cartagena	234	56.8
**Total**	412	100.0
**Individual life stage (Mean 40.27; 18–65 years)**		
Youth	91	22.1
Adulthood	264	64.1
Older adulthood	55	13.3
**Marital status**		
Married	77	18.7
Separated	22	5.3
Cohabiting	183	44.4
Single	121	29.4
Widowed	9	2.2
**Gender**		
Male	79	19.2
Female	333	80.8
**Educational level**		
None	13	3.2
Incomplete primary	40	9.7
Completed primary	27	6.6
Incomplete secondary	84	20.4
Completed secondary	136	33.0
Technical	90	21.8
University	22	5.3

**Table 2:** Descriptive statistics for well-being scales Colombian Caribbean Region (Departments of Atlántico and Bolívar), 2023–2024.

Scale	Dimension	Mean	SD	Min	Max
Ryff’s Psychological Well-being	Self-acceptance	23.03	5.12	8	30
(Likert scale from 1 to 6)	Positive relations	15.67	4.87	4	24
	Autonomy	24.79	5.48	11	36
	Environmental mastery	23.12	5.12	7	30
	Personal growth	16.87	4.05	4	24
	Purpose in life	24.53	5.49	5	30
Keyes’ Social Well-being	Social integration	18.04	4.48	5	25
(Likert scale from 1 to 5)	Social acceptance	15.29	5.58	6	30
	Social contribution	19.92	4.79	5	25
	Social actualization	16.54	3.76	8	25
	Social coherence	11.94	4.06	4	20
Diener’s Subjective Well-being (Likert scale from 1 to 5)	Life satisfaction	17.12	5.49	5	25
Positive and Negative Affect (PANAS) (Likert scale from 1 to 5)	Positive affect	13.84	5.20	0	26
	Negative affect	22.54	5.80	0	30
Perceived Social Support	Family support	22.53	7.22	0	28
(Dichotomous scale 0–7)	Friends support	21.11	7.37	0	28
	Significant others support	17.93	8.89	0	28

**Table 3:** SRQ scores Colombian Caribbean Region (Departments of Atlántico and Bolívar), 2023–2024.

	Barranquilla		Cartagena	
	N = 178	%	N = 237	%
**Mental Health Problems**				
Absence	162	91.0	203	86.4
Presence	16	9.0	31	13.2
**Psychosis**				
Absence	65	36.5	35	14.9
Presence	113	63.5	199	84.7
**Alcohol-related Problems**				
Absence	166	93.3	186	79.1
Presence	12	6.7	48	20.4

**Table 4:** Family functioning and burden. Caribbean Region of Colombia (Departments of Atlántico and Bolívar), 2023–2024.

Zarit Scale	N	Percentage
No overload	34	19.1
Mild overload	3	1.7
Severe overload	11	6.2
**Family APGAR**		
Good functioning	191	46.2
Mild dysfunction	106	25.7
Moderate dysfunction	30	7.3
Severe dysfunction	44	10.7
Missing data	41	10.2

**Table 5:** Spearman’s Rho Correlations. Colombian Caribbean Region (Departments of Atlántico and Bolívar), 2023–2024.

	Mental Health Screening	Mental Health Problems	Psychosis	Alcoholism	Risk of Seizure Disorder
Self-acceptance	0.016	−0.042	0.170 *	0.136	−0.053
Positive Relations	−0.075	−0.105	−0.006	0.033	−0.103
Autonomy	0.075	0.022	0.158 *	0.135	−0.108
Environmental Mastery	0.084	0.023	0.189 *	0.088	−0.027
Personal Growth	0.097	0.050	0.226 **	0.027	−0.072
Purpose in Life	0.147	0.077	0.296 **	0.054	−0.031
Social Integration	0.189 *	0.110	0.299 **	0.146	−0.057
Social Acceptance	−0.405 **	−0.362 **	−0.353 **	−0.024	−0.055
Social Contribution	0.155 *	0.064	0.338 **	0.132	−0.007
Social Actualization	−0.055	−0.106	0.112	0.133	0.122
Social Coherence	−0.292 **	−0.279 **	−0.217 **	0.059	−0.073
Subjective Well-being	−0.215 **	−0.256 **	−0.037	0.091	−0.008
Positive Emotions	0.182 *	0.244 **	−0.033	−0.118	0.040
Negative Emotions	−0.181 *	−0.213 **	−0.072	0.079	−0.062
Support from Significant Others	0.000	−0.040	0.112	0.064	−0.164 *
Family Support	−0.138	−0.167 *	−0.024	0.050	−0.064
Friends Support	−0.212 **	−0.208 **	−0.124	−0.007	−0.101
Caregiver Burden	−0.290 **	−0.268 **	−0.349 **	0.069	0.028
Family Functioning	−0.188 *	−0.178 *	−0.163 *	−0.069	0.013

* Correlation is significant at the 0.05 level (two-tailed). ** Correlation is significant at the 0.01 level (two-tailed).

**Table 6:** Bivariate analysis of risk (low/high) and protective factors for mental health problems.

Variable	Category	Risk (n) High	Low	Sig.
Family structure	Nuclear	59	1	<0.001
	Extended	64	4	
	Expanded	10	1	
	Single-parent	8	7	
	Reconstituted	13	3	
	Conjugal dyad	6	0	
	Single-person	2	0	
Alcohol consumption	No	142	12	0.157
	Yes	20	4	
Tobacco consumption	No	159	14	0.014
	Yes	3	2	
Other substance use	No	160	16	0.655
	Yes	2	0	
Separation	No	150	12	<0.001
	Yes	12	2	
	Yes, ongoing	0	2	
Unemployment	No	88	6	0.244453
	Yes	48	8	
	Yes, ongoing	26	2	
Relationship problems	No	146	9	<0.001
	Yes	12	7	
	Yes, ongoing	4	0	
Support from significant others	Absent	11	9	<0.001
	Present	151	7	
Family support	Absent	19	8	<0.001
	Present	143	8	
Friend support	Absent	44	11	<0.001
	Present	118	5	
Zarit scale	No burden	31	3	0.021
	Mild burden	2	1	
	Severe burden	6	5	
Family APGAR	Good functioning	63	2	<0.001
	Mild	56	3	
	Moderate	12	3	
	Severe	11	5	

**Table 7:** Multiple linear regression model for mental health problems (n = 370).

Variable	B	Std. Error	Standardized β	t	*p*	VIF
Gender (female = 1)	1.50	0.48	0.141	3.13	0.002 *	1.06
Family APGAR	−0.145	0.048	−0.156	−3.05	0.002 *	1.38
Social Acceptance	−0.071	0.040	−0.089	−1.78	0.076	1.32
Social Coherence	−0.228	0.052	−0.223	−4.41	<0.001 *	1.34
Family Structure	0.040	0.118	0.015	0.34	0.733	1.03
City (Cartagena = 1)	1.63	0.55	0.192	2.98	0.003 *	2.19
Relationship Problems (yes)	1.94	0.48	0.187	4.05	<0.001 *	1.12
Negative Emotions	0.134	0.038	0.228	3.56	<0.001 *	2.16
Friend Support	−0.076	0.026	−0.140	−2.92	0.004 *	1.22
Family Support	−0.080	0.030	−0.142	−2.63	0.009	1.54

* Significant after FDR correction.

**Table 8:** Binary logistic regression model for mental health problems (n = 370).

Variable	B	Std. Error	OR (95% CI)	*p*
Gender (female vs. male)	0.25	0.58	1.29 (0.42–3.98)	0.663
Family APGAR (scale)	−0.07	0.04	0.93 (0.86–1.01)	0.096
Social Acceptance	0.02	0.04	1.02 (0.93–1.11)	0.724
Social Coherence	−0.23	0.06	0.80 (0.71–0.90)	<0.001 *
City (Cartagena vs. Barranquilla)	1.42	0.57	4.13 (1.37–12.5)	0.012 *
Relationship Problems (yes)	1.54	0.43	4.67 (2.03–10.7)	<0.001 *
Negative Emotions	0.11	0.04	1.12 (1.04–1.21)	0.005 *
Friend Support	−0.06	0.03	0.94 (0.90–0.99)	0.033
Family Support	−0.04	0.03	0.96 (0.91–1.01)	0.127

* Significant after FDR correction.

## Data Availability

The original data presented in the study are openly available in https://osf.io/k72ts, 4 September 2025, https://doi.org/10.17605/OSF.IO/M9KVJ.

## Supplementary Materials

The following supporting information can be downloaded at: https://www.mdpi.com/article/10.3390/jcm15041416/s1, Table S1. STROBE checklist.
