# Supplementary material for: Psychological Well-Being and Mental Health in Caribbean Communities in Light of the Methodological Triangulation of the Classical Approach and Network Analysis
Source: J Clin Med. 2026 Feb 11;15(4):1416. doi: 10.3390/jcm15041416 (PMC12941793; doi:10.3390/jcm15041416)
Supplement: Supplementary file 1 [file jcm-15-01416-s001.zip › jcm-3886004-supplementary.pdf]

Table S1. STROBE checklist

STROBE Section / Item	Compliance in this study	Location in the manuscript
Title and abstract: Indicate the study design in the title or abstract	Yes, reported in the title and abstract	1
Background/rationale: Explain the scientific background and rationale for the study	Met	2
Objectives: State specific objectives and any prespecified hypotheses	Met	3
Study design: Present key elements of study design early in the paper	Met	4
Setting: Describe the setting, locations, and relevant dates, including periods of recruitment, exposure, follow-up, and data collection	Met	4
Participants: Give eligibility criteria, and the sources and methods of selection of participants	Met	4
Variables: Clearly define all outcomes, exposures, predictors, potential confounders, and effect modifiers	Partial (missing details on operationalization)	5
Data sources/measurement: Describe sources of data and methods of assessment (measurement)	Met	5
Bias: Describe any efforts to address potential sources of bias	Met	4
Study size: Explain how the study size was arrived at	Partial, due to the nature of the sampling	4
Quantitative variables: Explain how quantitative variables were handled in the analyses	Met	6
Statistical methods: Describe all statistical methods, including those used to control for confounding	Met	6
Participants: Report numbers of individuals at each stage of the study	Met	4
Descriptive data: Give characteristics of study participants (e.g., demographic, clinical, social)	Met	4
Outcome data: Report numbers of outcome events or summary measures	Met	7-16
Main results: Give unadjusted estimates and, if applicable, confounder-adjusted estimates and their precision (e.g., 95% CI)	Met	6-12
Other analyses: Report other analyses done—e.g., analyses of subgroups and interactions	Met	12-16
Discussion: Summarize key results with reference to study objectives	Met	16-18
Limitations: Discuss limitations of the study, taking into account sources of potential bias or imprecision	Met	18
Interpretation: Give a cautious overall interpretation of results considering objectives, limitations, multiplicity of analyses, results from similar studies, and other relevant evidence	Met	16-18
Generalisability: Discuss the generalisability (external validity) of the study results	Met	19-20
Funding: Give the source of funding and the role of the funders	Met	20
